# Assessment of knowledge of malaria and its control practices in mining and sugarcane growing regions of Western Kenya highlands

**DOI:** 10.4314/ahs.v22i2.23

**Published:** 2022-06

**Authors:** Davis Kipcho Mukabane, Nicholas Kitungulu, Philip A Ogutu, Jackson Cheruiyot Korir, David Hughes Mulama

**Affiliations:** Biological Sciences Department, MasindeMuliro University of Science and Technology, P.O. Box 190-50100, Kakamega, Kenya

**Keywords:** Malaria, control practices, Kenya highlands, Mining

## Abstract

**Background:**

Despite upscaled control efforts, deaths and hospitalization due to malaria remained high in counties of western Kenya highlands.

**Objectives:**

This study assessed the knowledge of malaria in two rural communities, the control strategies they use, and their capacity to integrate the available control programs.

**Methods:**

A cross-sectional survey was carried out in two rural villages in November – December 2018. Focus group discussions and a questionnaire survey were carried out in 736 households. Frequencies and proportions were used for descriptive analysis while the Chi-square test was used to determine factors that were associated with knowledge of malaria at p ≤ 0.05

**Results:**

Ninety-seven percent of the respondents had knowledge of malaria and this was associated with the level of education attained (χ2 = 30.108; p > 0.0001). Bed net ownership was at 86% and 92% correctly identified its use. Draining stagnant water (53.9%) was the most cited environmental management practice.

**Conclusion:**

There was awareness of the risk factors of malaria transmission in the study sites. The local communities must be mobilized and empowered through EIC for the control practises to bear fruit against malaria transmission. However, more sensitization needs to be done to optimize the use of malaria control practices.

## Introduction

Malaria is still reported in many areas around the world even with concerted efforts to control the transmission. In 2016 Sub-Saharan Africa (SSA) reported 90% of the cases^1^. In Kenya, the control practices are widespread in malaria-endemic zones where malaria prevalence remains high^2^. Despite these control efforts, deaths from malaria and hospitalization due to the disease are still high especially in highlands west of the Rift Valley^3^. This suggests that there could be a stagnation in the fight against the malaria scourge. This calls for newer and more effective control tools and practices to shore up the existing ones without reversing the gains already made. The focus has been more on the long-lasting insecticidal nets (LLINs) and IRS. Other strategies can be incorporated and implemented at the individual household level to significantly limit human-vector interaction^4^. Besides environmental management and bed net use, perceptions and knowledge of the people enhance efforts to eliminate the vector^5^.

The socio-economic abilities of individuals and populations have a crucial role to play in the malaria cycle^6^. Poverty is a major cause of unnecessary deaths due to malaria and other public health issues^7^. Economic status determines the success or failure of a program by an individual or government since impoverished households would not consume services that would otherwise protect them against the malaria burden. Artisanal gold mining and farming are socio-economic activities that alter the landscape and have been implicated in malaria transmission^8^,^9^ Besides, artisanal mining is associated with the poor^10^ whereas sugarcane farming puts a constraint on available farmland available for other activities^11^. Available data suggest that the poorest people remain least likely to adopt recommended disease control practices and get treatment. Factors that prevent access to this treatment are not well documented^12^. Therefore, poorly managed water holding pans such as mines, fish ponds, burrow pits, and drainages in Rosterman mines and Eluche regions create ideal breeding habitats for Anopheles, thus contributing to malaria transmission. A previous study in the two sites implicated the availability of aquatic habitats to the proliferation of Anopheles^13^. Malaria remains a major public concern in the two sites and other parts of the Western Kenya highlands^14^. The missing link between the resurgence of malaria in the Western Kenya highlands and these economic activities requires investigation to assess the success of the control programs available.

People of the same economic standing live in similar house settings^15^. Poorly constructed dwellings enable the mosquito vector to gain access to the dwellers16 through cracks in walls/roofs, eaves, and unscreened windows and doors. Entry of the vector into human dwellings is also because of the bodily odors and heat generated from within17. Houses that are squeezed with more occupants attract more of the vector than spacious ones with few occupants^18^. Additionally, it has been reported that living space for poor families may not be well separated from the domestic and that this animals' body heat also attracts mosquitoes^19^. Therefore, structural factors complicate the control practices put in place by many households. Dwellings with more members sleeping together are characteristic of poverty levels and even with the availability of the mosquito net, proper use becomes another challenge^20^.

Despite ownership of the bed net rising to above 80% in the last decade^21^ malaria cases have remained high. Under the universal coverage and usage in SSA, the net is meant to prevent host-vector interaction by preventing access of the nocturnal anopheles vector, repelling or killing it. However, a gap exists between possession and proper use of bed nets^20^. Therefore, an effort is required to change people's behavior by providing accurate information. Besides the net, other control practices including traditional methods^16^ have to be integrated to reduce the transmission of malaria. Household and community ability to implement and sustain these malaria control strategies are of great significance to the rollback malaria initiative. Little is available on the knowledge, attitudes, and practices (KAP) among populations in mining and sugarcane growing areas about malaria and the control methods they employ in the prevention of the disease. This study endeavored to find out this missing link in the fight against malaria in two rural communities of Western Kenya highlands. The findings will give crucial information to authorities on the success of malaria control programs based on how the communities consider malaria to be a public health concern to them and if they have the skills and knowledge to participate in its prevention.

## Methods

This was a pilot cross-sectional study carried out in November – December 2018 as part of a wider study. Rosterman mines (Latitude 0.2833°N, Longitude 34.7500°E) at 1400 - 1500 meters above sea level (asl) and Eluche (Latitude 0.33511°N, Longitude 34.4864°E) at 1300 - 1400 meters asl, are two rural villages in Kakamega county, western Kenya. The communities' economic activities include mixed farming and rearing livestock, artisanal mining, sand harvesting, and small-scale business. These land modifying activities create water-holding pits in which Anopheles breed and hence sustain malaria transmission in the population^13^. Focus group discussions (FGDs), and a questionnaire survey were used. FGDs were composed of 3 to 7 members who were purposely selected to include village elders, opinion leaders, and community health volunteers (CHVs), and a moderator from the research team. Each group met for one hour, three times during the study period. Participants who were adult Kenyans of both sexes, residents of the two areas, and those willing to contribute, were included in the FGDs. Since the FGDs' themes were open and not gender or culturally sensitive, the data collected from them was not confidential. The questionnaire sought to find out: socio-demographic characteristics of the households, knowledge of the household head about malaria (source of information about malaria, cause, and transmission), mosquito breeding sites, preventive measures (short and long term), and availability of the net and its use. It was pre-tested, translated to the local dialect, and was verbally translated to Kiswahili to those who had difficulties with English or local dialect. The interviewers were trained on approaching, consenting, and filling the questionnaire. This was systematically done house to house giving a total of 736 households. For quality control, a supervisory meeting was held between the principal researchers and the field team after each session.the sessions were used for standardization of responses, clarity of expectations, and conformation of responses.

### Data management and analysis

Collected data were entered in MS Excel spreadsheet, checked and cleaned of errors and inconsistencies. After which it was coded and processed using statistical package for social sciences (SPSS) version 20. Frequencies and proportions were used for descriptive analysis of the data. Logistic regressions were done for selected risk factors of interest. Odds ratios (OR) and the Chisquare tests were computed to determine the strength of association at 95% CI and p ≤ 0.05. Data collected from FGDs were observational and qualitative.

### Ethical considerations

Ethical clearance to undertake the study was granted by MasindeMuliro University of Science and Technology Institutional Ethical Review Committee (IERC) vide approval number MMUST/IERC/090/19. Permission to proceed with the study was given by National and Kakamega County administrative authorities. The participants signed the coded consent forms before proceeding to fill the questionnaires after the objective and methodology had been explained to them. The coding and filling was done in the participants homes for confidentiality purposes. Responders were informed that there was no direct monetary benefit and absence and the unwillingness of a resident to participate excluded them from the process.

## Results

### Socio-demographic characteristics of the participants

The socio-demographic variables were distributed among participants as shown in Table 1. Of the participants in both FGDs and interviews, 56.5% of the cumulative total were female, 70.2% were married, 39.8% attained post-primary education, and 32.5% were in formal and informal employment.

**Table 1:** Socio-demographic characteristics of the household heads surveyed

Variable	Category	Frequency		Percent		Cumulative Total	
		Rosterman		Eluche		N	%
		
		N	%	N	%		
Gender	Male	191	50.3	129	36.2	320	43.5
	Female	189	49.7	227	63.8	416	56.5
Marital status							
	Unmarried	74	19.5	49	13.8	123	16.7
	Married	256	67.4	261	73.3	517	70.2
	Widowed	43	11.3	44	12.4	87	11.8
	Divorced	7	1.8	2	0.6	9	1.2
Level of education							
	No education	48	12.6	62	17.4	110	14.9
	Primary	160	42.1	173	48.6	333	45.2
	Secondary	129	33.9	101	28.4	230	31.2
	Tertiary	43	11.3	20	5.6	63	8.6
	Others						
Occupation							
	Self-employed in agriculture	72	18.9	91	25.6	163	22.1
	Self-employed in business	85	22.4	57	16.0	142	19.3
	Housewife	82	21.6	37	10.4	119	16.2
	Employed	101	26.6	138	38.8	239	32.5
	Unemployed	40	10.5	33	9.3	73	9.9

### Assessment of household's head knowledge about malaria

To assess the KAP of the head of the household on malaria, seven questions on the source of information about malaria, cause of malaria, mode of transmission, methods of prevention, mosquito breeding sites, signs of malaria, and the mosquito net were asked. Participants who gave at least three correct responses to the first five key questions were considered knowledgeable enough. Ninety-seven percent (n = 719) of the total respondents admitted to having information about malaria with the radio/television the most reported source. The role of community health workers (CHVs) was highly pronounced in the FGDs:

“*Following the increase of CHVs in our village, we now have people to ask questions about malaria and other illnesses*,” said Rosterman village pastor.

Plasmodium (52.9%) was the most cited cause of malaria followed by germs (17.7%), bites by the mosquito (17.0%), and dirty stagnant water (4.8%) [Table 2]. There was an association between socio-demographics and knowledge of the cause of malaria (χ2 = 30.108; p > 0.0001). On the transmission of the malaria parasite, a bite by any mosquito was 51.0% followed by a bite of a mosquito that had bitten a malaria patient at 33.8%. There was a significant association between socio-demographics and knowledge of malaria parasite transmission (χ2 = 15.663; p = 0.001). About knowledge of mosquito breeding sites, 55.0% cited bushes followed by others (water-holding containers, tanks) at 25.1%, tall grass 12.6%, and stagnant water 7.2%. There was a significant association between socio-demographics of respondents and knowledge of the mosquitoes breeding site (χ2 = 17.696; p > 0.0001).

**Table 2:** Respondents' knowledge on the cause of malaria, transmission, and breeding sites of the vector

Variables		Rosterman		Eluche			
	
	Response	N	%	N	%	χ^2^	*P*-value
Cause of malaria	Germs	73	19.2	57	16.3	30.11 (3df)	0.000
	Dirty stagnant water	24	6.3	11	3.1		
	Mosquito bites	82	21.6	43	12.1		
	*Plasmodium*	185	48.7	204	57.3		
	Does not know	16	4.2	41	11.5		
Ways of parasite entry	By bites of any mosquito	204	53.7	171	48.0	15.66 (3df)	0.001
	By bites of mosquito which has bitten a malaria patient	111	29.2	138	38.8		
	Others	41	10.8	17	4.8		
	Do not know	24	6.3	30	8.4		
Mosquito breeding sites	Stagnant water	41	10.8	12	3.4	17.70 (3df)	0.000
	Tall grass	53	13.9	40	11.2		
	Bushes	199	52.4	206	57.9		
	Others	87	22.9	98	27.5		

**Figure 1:**
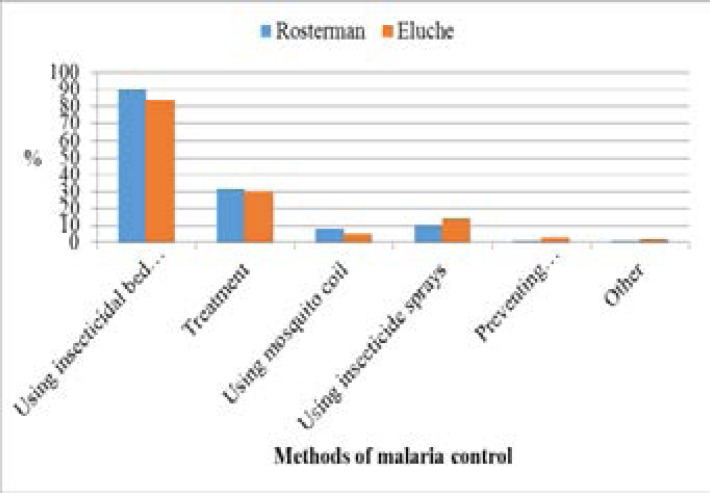
Respondents' communicated malaria control methods

Over 95% of the respondents were able to identify at least three symptoms of malaria. They correctly identified shivering, headache, sweating, vomiting, loss of appetite, joint pains, high fever, and feeling cold as the most notable symptoms of malaria that compelled them to seek medical assistance or buy antimalarials. The majority of those interviewed had proper knowledge about how to prevent malaria, with the bed net the most mentioned method [Figure 1]. However, in the FGDs it was reported that mosquitoes bit them at odd hours:

“*The mosquitoes bite when we are seated out selling our wares like vegetables or when milking or in the kitchen cooking*,” said Mama Mboga from Eluche

However, fears about net use arose particularly from members of FGDs where they reported that they feared the bed nets would catch:

“Our bed nets can catch fire from the cooking place adjacent to the bed or from the oil lamps we use. This puts us in a dangerous situation,” opined a village elder from Rosterman.

**Figure 2:**
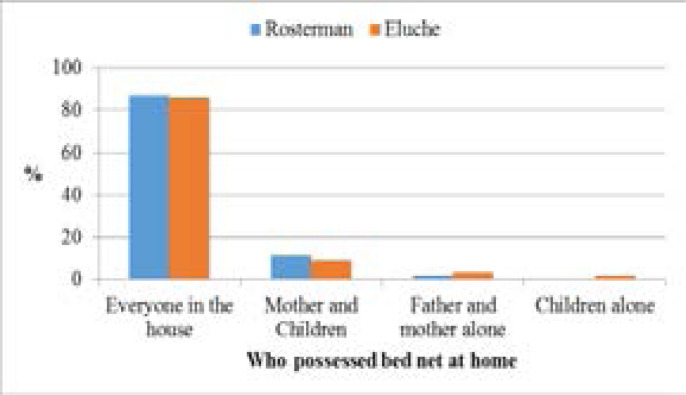
Respondents' net ownership

**Figure 3:**
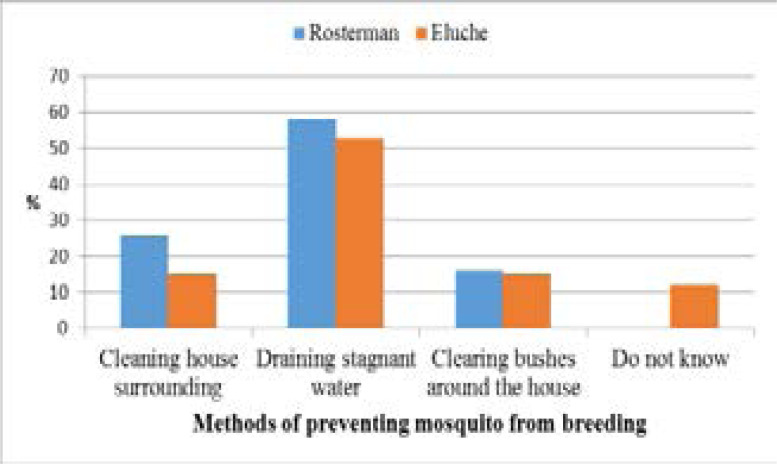
Known and Used Environmental Management Methods

Figure 2 shows Net ownership in sampled households. Ninety-seven percent of the homesteads with the bed net had the long-lasting insecticide-treated (LLIN) type which was bought, given by health campaigners, or given at health centers (during neonatal or postnatal care). In both study sites, preventing breeding/resting places for mosquitoes received the second least responses despite featuring prominently in known environmental management methods in malaria control [Figure 3]. Draining stagnant water was the most used method outdoors to prevent the mosquito from breeding and as a result control malaria transmission. Level of education played a significant role (p = 0.046) and gender being of no notable significance (p = 0.229) in malaria control.

An observed challenge to proper environment management aimed at reducing mosquito breeding sites was the socio-economic activities of the two communities. This was addressed by a member in one of the FGDs.

“We are faced with a dilemma about draining stagnant water and our daily gold mining activities. The gold mines are our sources of our daily food and we may not fill them” opined the village elder in Rosterman.

Regression analysis showed that demographic factors had no impact on the knowledge about methods of malaria prevention and control as summarized in Table 3. Ownership and use of the bed net, the choice of the environment management practice employed by a household, and applying IRS were in no way associated with socio-demographic characteristics.

**Table 3:** Demographic variables and their impact on knowledge about methods of malaria prevention and control

Variables	N	Odds Ratio	95% CI	*P value*
**Factors associated with** **knowledge of bed nets**				
Age				
≤19	77	0.114	0.012–1.062	0.056
20–39	340	0.275	0.026–2.924	0.284
40–59	205	0.485	0.485–4.4745	0.534
≥60	114	1.000	0.031–4.123	0.271
Education				
No education	110	1.311	0.127–13.498	0.820
Primary	333	1.166	0.138–9.824	0.888
Secondary	230	1.238	0.140–10.959	0.848
Tertiary qualifications	63	1.000	0.171–9.628	0.812
**Factors associated with** **knowledge of malaria**				
Gender				
Male	320	0.782	0.518–9.212	0.287
Female	416	-0.782	0.109–1.929	0.287
Education				
No education	110	17.205	0.000	0.848
Primary	333	16.467	0.000	0.996
Secondary	230	0.000	0.000	0.997
Tertiary qualifications	63	1.000	0.000	0.837
**Factors associated with** **environmental management**				
Gender				
Male	320	-0.015	0.555–1.750	0.959
Female	416	0.015	0.959–1.015	0.959
Education				
No education	110	1.930	0.868–54.680	0.068
Primary	333	1.699	0.730–41.013	0.098
Secondary	230	1.228	0.435–26.761	0.243
Tertiary qualifications	63	1.318	0.623–28.217	0.378

## Discussion

The FGDs were good sources of information about knowledge of malaria and control practices employed in the study sites. Participants attributed transmission to a confluence of numerous factors among them was lack and inappropriate use of personal protection during the night. In the FGDs participants reported that they were normally bitten by mosquitoes while in the house during supper time when watching the news, or chatting before they slept and during sleeping. This corroborates findings of other studies in Nyabondo, Kenya^16^, and Yaounde, Cameroon^22^. The Anopheles mosquito is an efficient vector and has adapted unique behavior enabling it to feed on humans while in their dwellings^23^ or during their evening chores outside. Findings in Equatorial Guinea^24^ attributed this to indoor interventions like spraying with insecticides and the LLINs. The findings showed that the communities acknowledge that malaria remains a major threat on life.

The findings showed that education and economic levels had a significant association with knowledge of malaria, its transmission, and methods employed to control it. This supported other studies carried out in South Africa^25^, where individuals with low economic status had suffered from malaria in the past. In the Democratic Republic of Congo (DRC), a study showed a similar trend in which people in dire economic status and rural areas were at a higher risk of getting infected than other groups^26^.

In the present study, respondents acknowledged having prior information about malaria and that they were only recipients and not formulators of information about malaria control. A variety of sources were reported including radio/television, health workers, family/friends, school, religious/administrative meetings, and print media. The reception of information from multiple sources supports another study done elsewhere^27^. The knowledge gained by the responders was immense in understanding the interaction between the disease, the vector, the environment, and the human population. In one FGD, it was observed that the community did not participate in the formulation and ownership of the control programs or messages about malaria but they were only recipients.

Having relevant EIC about malaria is a major cornerstone to its control. This study found out that both providers and recipients of information about malaria had relevant knowledge about appropriate control methods at a personal and communal level. The bed net ownership was above the national average and its use was reported by the majority of respondents. This corroborates findings from another study in which the bed net reduced interaction between the human and the vector during sleep^28^. Appropriate net use goes a significant way to reduce malaria bouts among people. The availability of the net in both regions was attributed to government and Non – G/span>overnmental Organizations' campaigns in the distribution. Getting the LLIN by the respondent was dependent on the availability of the respondent during the time of distribution. Additionally, the net was given to expectant mothers and children under 5 years during visits to the health facilities. However, it can be reported that the high LLIN availability and use did not match the knowledge of the transmission and prevention strategies for malaria by respondents. This scenario has also been reported in another study in Bangladesh^29^. However, it can also be reported that knowledge uptake can be hampered by indecision or misinformation. For instance, in FGDs members said they feared the nets could easily catch fire from the cooking place adjacent to the bed or the oil lamps they use as a source of light. Therefore, the community health workers must work in homes and assist in teaching the respondents on aspects like hanging and using the LLIN^30^. Proper use of the net is critical in reducing transmission of malaria and comprehensive information should reach the communities regularly. Other personal protection methods such as the use of mosquito coil, repellents, or spraying were sparsely mentioned by the respondents or during the FGDs. This could be attributed to a lack of proper awareness and communication. This has been reported in another study as a possible limiting factor to the success of reducing malaria transmission^16^.

Knowledge of the vector and proper use of vector control methods is very crucial in cutting transmission^16^. Availability of mosquito breeding grounds like derelict mining pits abandoned fish ponds, and poorly managed drainages that are too close to human dwellings enhance malaria transmission. Human and natural, permanent, and localized pits that hold water are known sources of vector all year round^31^. This study reported that there is higher awareness and use of appropriate environmental management interventions at the individual household level. In as much as draining stagnant water, clearing tall grasses and bushes, and keeping the environment around the house clean were reported to reduce considerably the number of mosquitoes available at a particular time or season, man-made and permanent water bodies in the study sites may drag the success of these interventions in combating malaria transmission as reported in FGDs. This contradicts another study that indicated that these practices are ineffective in reducing populations of Anopheles unless there is inter-sectoral collaboration in the management of the environment^32^. The success of these methods depends on the feeding characteristics of the species of Anopheles found in the study sites. Endophilic Anopheles rest indoors and therefore defeat the use of these malaria control practices^33^. In this, FGDs members noted that mosquito numbers are highest when maize plantations are blossoming and every available space is green with vegetation. This indicated a lack of knowledge that this occurrence coincided with the rainy season when breeding sites are readily avilable. As was also reported by a study in Brazil^34^. The success of environmental management methods of malaria control depends on engaging all the households. For instance, draining of stagnant water would be defeated if some households do not participate because mosquitoes would breed in the stagnant water in their jurisdiction. The findings of this study suggested that these methods must be owned and maintained through participation and sustained through continuous education to effectively mitigate malaria transmission.

## Conclusion and Recommendations

This study reported awareness of the risk factors of malaria transmission in the two rural villages at both household and community levels. Knowledge about the connection between socio-demographic factors, environmental management, mosquito breeding, and resting sites, and malaria transmission is key in fore-stalling malaria transmission. The local communities must be mobilized and empowered through EIC for the control practises to bear fruit against malaria transmission. Therefore, efforts should be channelled at including as many people as possible in the fight against malaria to embrace individual household and communal participation. This study recommended that during education promotions and sensitization campaigns about malaria, emphasis should be laid on educating recipients on integrated control activities that result in a reduction in mosquito density enabling proper use of an individual's time and meager resources.
